# The neural control of singing

**DOI:** 10.3389/fnhum.2013.00237

**Published:** 2013-06-03

**Authors:** Jean Mary Zarate

**Affiliations:** Department of Psychology, New York UniversityNew York, NY, USA

**Keywords:** auditory processing, audio-vocal integration, dual-stream model, non-musicians, singers, somatosensory, vocal pitch

## Abstract

Singing provides a unique opportunity to examine music performance—the musical instrument is contained wholly within the body, thus eliminating the need for creating artificial instruments or tasks in neuroimaging experiments. Here, more than two decades of voice and singing research will be reviewed to give an overview of the sensory-motor control of the singing voice, starting from the vocal tract and leading up to the brain regions involved in singing. Additionally, to demonstrate how sensory feedback is integrated with vocal motor control, recent functional magnetic resonance imaging (fMRI) research on somatosensory and auditory feedback processing during singing will be presented. The relationship between the brain and singing behavior will be explored also by examining: (1) neuroplasticity as a function of various lengths and types of training, (2) vocal amusia due to a compromised singing network, and (3) singing performance in individuals with congenital amusia. Finally, the auditory-motor control network for singing will be considered alongside dual-stream models of auditory processing in music and speech to refine both these theoretical models and the singing network itself.

Most of the literature on sensory-motor control in music production and training-induced plasticity focuses on trained instrumental musicians or learning paradigms with musical instruments (e.g., learning to play short piano melodies, etc.). Singing, however, provides a unique opportunity to examine sensory-motor processes during musical production, since the instrument is already contained within the body; there is no need to create artificial instruments to assess motor control mechanisms with neuroimaging or any other experimental approach. Moreover, the adult vocal apparatus is highly trained to produce nuanced utterances in both song and speech. Across their lifetime, healthy non-musicians have sung (or have attempted to sing) a full repertoire of songs in socially and culturally specific settings, (“Happy Birthday,” their national anthem, etc.). Additionally, healthy individuals can control their vocal pitch and/or output intensity to indicate the intent of a sentence (e.g., declarative statements vs. questions vs. commands), set the emotional context for a conversation (e.g., happiness, anger, sadness), or in tonal languages, distinguish between words and their meanings. Singers, on the other hand, undergo many years of extensive sensory-motor training and practice to exert much finer vocal control during more difficult tasks, such as singing fast vocal runs (e.g., melismata, melodic embellishments, etc.) or maintaining a melodic passage as someone else simultaneously sings a harmonic line. Therefore, using singing tasks to test groups with different levels of singing experience is a rare opportunity to determine how musical experience specifically enhances sensory-motor control of this particular instrument, beyond the remarkable feats it already can perform. However, the mechanisms by which the vocal instrument is precisely controlled for singing are highly complex and thus require multiple networks for vocal motor control and sensory feedback processing.

## Sensory-motor control of vocalization

### Sensory-motor control observed from the vocal tract

When air passes through the glottis (opening of the larynx) and causes the vocal folds surrounding the glottis to vibrate at a particular rate, the resulting vibration rate determines the fundamental frequency (i.e., perceived pitch) of the voice (Sundberg, 1987). Different intrinsic and extrinsic laryngeal muscles interact to regulate fundamental frequency by altering the length of the vocal folds, thus changing the rate of vocal-fold vibration (Hirano et al., 1969; Sundberg, 1987). The precise control of laryngeal muscles is maintained in part by laryngeal reflexogenic control systems, in which receptors within the larynx adjust muscular contractions during perturbations. For instance, during vocalization, the uneven airflow passing through the glottis stimulates the myotatic mechanoreceptors in the intrinsic laryngeal muscles; these stretch-sensitive receptors initiate reflexive muscular adjustments to ensure that the vocal folds remain at the intended length and tension and therefore maintain a steady vocal pitch (Wyke, 1974). Additional reflexogenic systems work in concert with the intrinsic laryngeal reflexogenic system to ensure a stable vocalization (Wyke, 1974). Vocalization also involves the coordination of many other muscles, including the diaphragm and abdominal/thoracic muscles to provide airflow and regulate vocal output intensity, and articulatory muscles (e.g., lip, jaw, and tongue muscles, Hardcastle, 1976; Sundberg, 1987). The articulatory muscles contain somatosensory receptors that play a role in generating different vocal-tract configurations, which shape the formant frequencies that contribute toward vowel formation and vocal timbre (Sundberg, 1987; Jürgens, 2002; Perkell, 2012).

Similar to the somatosensory contribution to reflexogenic vocal control systems, auditory feedback also plays a role in reflex-like adjustments of ongoing vocal motor control. For instance, a slight *decrease* in auditory feedback amplitude elicits a quick *increase* in vocal output amplitude, which is known as the Lombard reflex (Lombard, 1911). During speech production, when the first formant frequency is shifted so that a produced vowel (e.g., /ε/) sounds like a different one (e.g., /æ/), the vocal motor system immediately compensates for the formant shift (Houde and Jordan, 1998, 2002; Purcell and Munhall, 2006a,b). Arguably, the most relevant auditory-vocal motor correction for singers deals with vocal pitch. When the pitch of auditory feedback is shifted up or down as participants vocalize for a few seconds (either at a comfortable pitch or to match a target pitch), investigators have observed pitch-shift responses, during which vocal pitch is adjusted quickly in the opposite direction of the feedback shift (Anstis and Cavanagh, 1979; Burnett et al., 1998; Larson, 1998; Hain et al., 2000; Jones and Munhall, 2000, 2005; Larson et al., 2000; Burnett and Larson, 2002; Liu and Larson, 2007; Jones and Keough, 2008). These pitch-shift responses often have two components: (1) an early pitch-shift response of 25–50 cents (irrespective of the pitch-shift magnitude) that occurs 100–150 ms after the pitch shift; and (2) a late pitch-shift response with a latency of 250–600 ms, whose magnitude and direction can be under voluntary control, if listeners are instructed to make a specific response (e.g., change pitch to either oppose or follow the pitch shift, etc., Burnett et al., 1998; Larson, 1998; Hain et al., 2000). Interestingly, prolonged exposure to feedback that is incrementally pitch-shifted over numerous trials can produce aftereffects in which intended vocal pitch and vocal output are mismatched, such that vocal pitch is automatically adjusted even when auditory feedback is returned to normal (Jones and Munhall, 2000, 2005; Jones and Keough, 2008).

### Neural networks governing sensory-motor control of vocalization

#### Brain regions involved in vocal motor control

**Figure 1:**
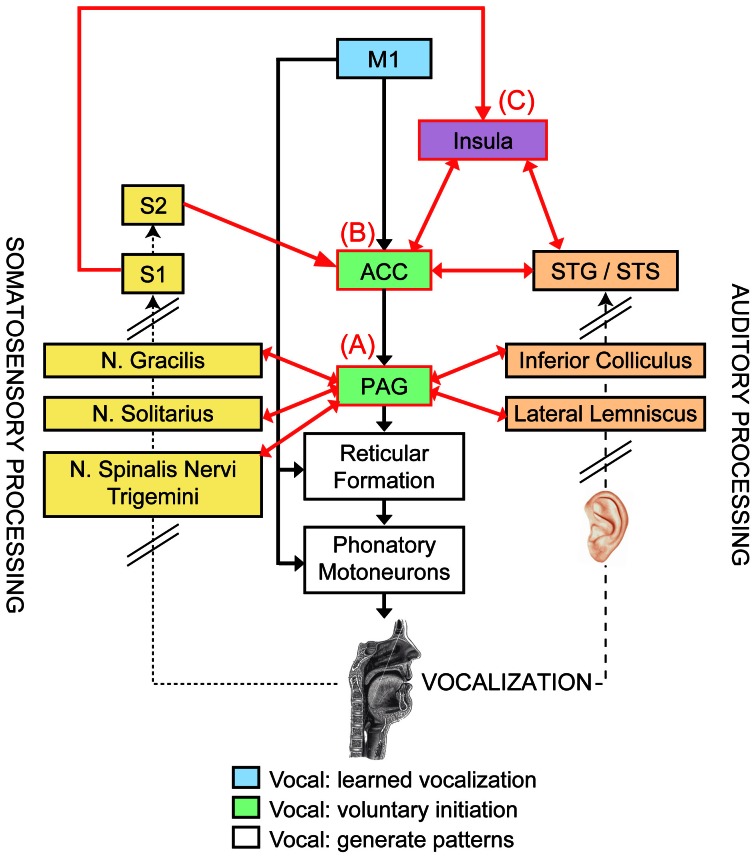
**Neural networks of vocal motor control (central column), somatosensory (left) and auditory feedback processing (right), and hypothesized regions of sensory-motor control of voice [modified from a model proposed by Jürgens (2009)].** The vocal motor control hierarchy starts with the generation of complete vocal patterns from the reticular formation and phonatory motoneurons (white boxes), and then the next highest level of control (green boxes) stems from the anterior cingulate cortex (ACC) and periaqueductal gray (PAG), which can initiate and emotionally motivate vocal responses. The highest level of vocal control comes from the primary motor cortex (M1, blue box; its modulatory brain regions are not depicted), which is responsible for producing learned vocalizations (i.e., speech and song). Somatosensory feedback (dotted arrow) from various receptors distributed throughout the vocal tract is processed in the ascending somatosensory pathway (yellow boxes, left; black slanted lines indicate that only selected regions of this pathway are shown) and transmitted to the primary and secondary somatosensory cortex (S1, S2). Auditory feedback (dashed arrow) from the vocalization is processed by the ascending auditory pathway and auditory cortical regions (orange boxes, right). Potential neural regions that integrate sensory feedback processing with vocal motor control are indicated with red-outlined boxes, and their shared connections are represented by red arrows: **(A)** the PAG, **(B)** ACC, and **(C)** the insula (in purple, classified as a higher-order associative area).

Multiple neural networks are required for precise control of the “phonatory” muscles mentioned above. The reticular formation of the pons and medulla has direct connections to the motoneurons for all phonatory muscles (Figure 1, white boxes, Thoms and Jürgens, 1987), and thus may coordinate phonatory muscle groups to generate complete vocal patterns (Jürgens and Hage, 2007). This region receives excitatory input from two distinct neural pathways of vocal control (Figure 1; Jürgens, 2009; Owren et al., 2011). The first vocal control pathway (Figure 1, green boxes) contains the anterior cingulate cortex (ACC) and the midbrain periaqueductal gray (PAG), both of which produce vocalizations when stimulated electrically or pharmacologically (Müller-Preuss and Jürgens, 1976; Müller-Preuss et al., 1980; Suga and Yajima, 1988; Dujardin and Jürgens, 2005). The second neural pathway includes the primary motor cortex (M1, Figure 1, blue box) and two subcortical loops—comprised of putamen, globus pallidus, pontine gray, and cerebellum—that modulate vocal motor commands from M1 and subsequently send modified motor programs via the ventrolateral thalamus back to M1; electrical stimulation of the ventral part of M1 elicits vocalizations, as well as individual movements of the jaw, tongue, and lips (Penfield and Rasmussen, 1950).

In humans, these networks form a tripartite hierarchy of vocal motor control (Figure 1, center column, Simonyan and Horwitz, 2011): (1) the reticular formation constitutes the lowest level at which complete vocal patterns are generated; (2) the next level is comprised of the ACC and the PAG, which are attributed with the voluntary initiation and emotional/motivational control of vocalizations (Jürgens, 2002, 2009); and (3) the highest level of vocal control occurs in M1 (and its modulatory brain regions), which is associated with the generation of learned vocalizations, such as speech and song (Jürgens, 2002, 2009). Importantly, this functional distinction of M1 is based on humans' unique possession of direct connections between the phonatory region of M1 (i.e., the ventral portion) and the motoneurons of phonatory muscles (see Figure 1); bilateral lesions to this M1 region destroys the ability to speak and sing (Jürgens, 2009), while innate vocalizations (e.g., shrieking, crying, etc.) that may be controlled by the ACC and PAG are left intact. In contrast, damage to the modulatory brain regions associated with M1 (e.g., putamen, globus pallidus, pontine gray, and cerebellum) can result in speech disorders such as stuttering and dysarthria (Ackermann et al., 1992; Jürgens, 2002; Alm, 2004). Lesions in the second level of vocal control may lead to mutism (attributed to PAG damage, Esposito et al., 1999) or loss of emotional/motivational intonation in speech (following damage to the ACC, Simonyan and Horwitz, 2011). Importantly, the functional organization of vocal motor control in humans is concurrently hierarchical and parallel, since damage to brain regions within the second or third levels does not abolish all vocalizations.

#### Neural processing of somatosensory feedback

Various somatosensory receptors transmit feedback about the current state of the vocal motor system (e.g., placement of articulators, respiration, etc.) via the glossopharyngeal and vagus nerves and the ascending somatosensory pathway, which includes the nuclei gracilis, solitarius, and spinalis nervi trigemini and the medial lemniscus in the medulla, and the ventral posteromedial nucleus in the thalamus (Jürgens and Kirzinger, 1985; Willis, 1986). The thalamus sends somatosensory information to primary and secondary somatosensory cortex (S1 and S2), as well as the insula (Jones and Powell, 1970; Augustine, 1996; Jürgens, 2002; Ackermann and Riecker, 2004, 2010). More specifically, the ventral portion of the primary somatosensory cortex (S1)—posteriorly adjacent to the M1 phonatory area that governs vocalizations and individual movements of the articulators (Penfield and Rasmussen, 1950)—processes somatosensory information about articulatory movements (Grabski et al., 2012), while the anterior portion of the insula is recruited particularly during overt vocalizations (compared to covert speech and song, Riecker et al., 2000) and may contribute to voluntarily controlled respiration during vocalizations in general (Ackermann and Riecker, 2010).

#### Neural processing of auditory feedback during singing

As each sung note reaches a singer's ear as auditory feedback, each of the different frequencies within that particular vocal pitch are transduced by the organ of Corti on the basilar membrane of the cochlea (Hudspeth, 2000). The frequency characteristics that are required to perceive the pitch are transmitted and/or processed along different parts of the ascending auditory pathway—comprised of the cochlear nucleus, lateral lemniscus, inferior colliculus, and the medial geniculate nucleus of the thalamus (Griffiths et al., 2001)—before the extracted frequencies (and many other attributes of sounds) are further processed in primary and secondary auditory cortex within Heschl's gyrus. In particular, pitch information may be processed specifically by a (rightward lateralized) pitch-sensitive area located in lateral Heschl's gyrus, reported to be involved in conscious pitch perception (Griffiths, 2003; Bendor and Wang, 2006). This region may also be involved in organizing pitches in a hierarchical fashion, since patients with lesions in this region displayed much higher discrimination thresholds than controls when asked to indicate the direction of pitch change between two notes (Johnsrude et al., 2000). Processing pitch changes or melodic phrases within a sung passage recruits additional auditory cortical regions outside of Heschl's gyrus, including regions in the right superior temporal gyrus (STG), planum polare, and planum temporale (Zatorre et al., 1994; Patterson et al., 2002; Hyde et al., 2008). When pitch comparisons are performed within a sequence of tones or short melodies, increased activity is observed within right auditory and frontal cortical regions presumably during tonal working memory processes, compared to passive melody perception (Zatorre et al., 1994). Melodic phrase comparisons in the same key, which may be done to ensure correct melodic reproduction, engages extensive activity within several auditory cortical regions along bilateral STG, whereas melodic phrase comparisons across a pitch transposition (i.e., a key change) engages additional activity from the intraparietal sulcus (IPS, Foster and Zatorre, 2010).

Aside from providing details about vocal pitch, auditory feedback can also provide information about vocal timbre, which is argued to be processed specifically along the superior temporal sulcus (STS, Belin et al., 2000). Kriegstein and Giraud (2004) discovered three functionally distinct regions along the STS. The anterior STS is associated with familiar voice recognition, while the mid/anterior STS preferentially responds to the spectral characteristics of voices. The posterior STS (pSTS), which is recruited during recognition of unfamiliar voices, may be involved in analyzing spectral details (or the changes therein) of voices over time (Kriegstein and Giraud, 2004; Warren et al., 2006). Given that the pSTS is also recruited in response to presentation of frequency-modulated sweeps of pure tones (Poeppel et al., 2004) and phonological processing (Hickok and Poeppel, 2007), this region may be involved generally in processing spectrotemporal fluctuations in sound, including notable changes in auditory feedback.

#### Potential substrates for integrating sensory feedback with vocal motor control

The constituents of the vocal motor network associated with voluntary initiation and emotional/motivational control of vocalizations—the PAG and ACC—receive both somatosensory and auditory input, and thus form two potential substrates for sensory-motor control of vocalization (Figure 1, red-outlined boxes and arrows). The PAG (Figure 1A) receives somatosensory input via afferent projections from the nucleus gracilis (implicated in respiratory control, Hannig and Jürgens, 2006) and nuclei solitarius and spinalis nervi trigemini (kinesthetic and proprioceptive information, Jürgens and Kirzinger, 1985; Yoshida et al., 2000), as well as auditory information from the inferior colliculus and lateral lemniscus (Dujardin and Jürgens, 2005), all of which may facilitate initiating vocalizations in response to external stimuli or adjusting vocalizations based on sensory feedback. For example, when connections to the cerebrum are severed, the Lombard reflex is preserved during PAG-induced vocalizations coupled with auditory masking, suggesting that the PAG may govern auditory-motor control during involuntary auditory-vocal reflexes (e.g., Lombard reflex, formant- and pitch-shift responses) without additional control from cortical regions (Nonaka et al., 1997). The ACC (Figure 1B) directly receives somatosensory input from S2 and auditory input from auditory cortical regions along the STG and STS (Jürgens, 1983; Barbas et al., 1999). This region also receives these types of sensory input indirectly from S1 and auditory association areas via the insula (Mesulam and Mufson, 1982; Augustine, 1996). Since the insula is a gateway of both somatosensory and auditory information for the ACC, this region itself may provide another substrate for sensory-motor control of vocalization (Figure 1C, purple box). In particular, the anterior insula, whose cytoarchitecture and projections classify it as an association area that integrates different modalities (e.g., auditory, visual, somatosensory, motor, etc., Rivier and Clarke, 1997; Lewis et al., 2000; Bamiou et al., 2003; Ackermann and Riecker, 2004), is engaged specifically during voiced speech and song, relative to covert or internal versions (Riecker et al., 2000; but see Hillis et al., 2004; Ackermann and Riecker, 2010 for conflicting clinical evidence of the insula's role in speech production).

#### Neuroimaging evidence: a general functional network for human vocalization

Neuroimaging studies from the past two decades have confirmed that many regions within vocal motor and sensory networks are recruited during various *overt* speech and song tasks, including: word or letter generation (Paus et al., 1993); syllable repetition (Riecker et al., 2005); singing a note repeatedly (Perry et al., 1999), in a sustained fashion (Zarate and Zatorre, 2008), or while changing vowels in particular rhythms (Jungblut et al., 2012); repeating syllables, spoken words, and sung or hummed melodies (Özdemir et al., 2006); humming, speaking, or singing lyrics of a well-known song (Formby et al., 1989; Jeffries et al., 2003); reciting the months of the year or singing a familiar melody (Riecker et al., 2000); telling a story (Schulz et al., 2005); improvising word phrases, melodies, or harmonies (Brown et al., 2004, 2006); spontaneous and synchronized speaking and singing (Saito et al., 2006); and singing an Italian aria (Kleber et al., 2007). Summarized from the neuroimaging evidence above, a general functional network for human vocalization (including speech and song) is comprised of the brain regions reviewed in the preceding sections: M1, ACC, basal ganglia, thalamus, and cerebellum for vocal motor control; S1 and S2 for somatosensory feedback processing; bilateral auditory cortical regions (primary auditory cortex and a pitch-sensitive region within Heschl's gyrus, various portions of STG and STS) for auditory feedback processing; and the insula presumably during multimodal processing of sensory feedback. In addition, premotor and parietal areas are recruited during human vocalization, and their functional roles will be further discussed below.

Until this point, both speech and song studies have been included to outline the brain regions associated with general vocal control in humans, since speaking and singing employ common mechanisms involved in vocal production. Moving forward, we will focus more on singing studies to examine how musical training modulates the general functional network for human vocalization as it is used for singing.

## Training effects on the sensory-motor control of singing

### Vocal training effects on the neural correlates of sensory-motor control of singing

In general, due to their extensive auditory-motor training and experience, musicians excel in various auditory and motor tasks. For instance, previous studies report that musicians perform better at pitch, timbre, and voice discrimination tasks than non-musicians (Kishon-Rabin et al., 2001; Tervaniemi et al., 2005; Chartrand and Belin, 2006; Micheyl et al., 2006). In addition to possessing better auditory discrimination skills than non-musicians, musicians also display more precise control over the vocal apparatus in the absence of proper auditory feedback. For example, trained singers sang more accurately with masked auditory feedback than non-musicians (Schultz-Coulton, 1978), yet one study reported the reverse (Watts et al., 2003). However, Watts' group of singers may have had less vocal training than the singers in Schultz-Coulton's study; Watts suggested that during the earlier stages of vocal training, more emphasis is placed on monitoring auditory feedback for vocal accuracy (Watts et al., 2003), which may account for their recruited singers' greater vocal inaccuracy with masked feedback compared to non-musicians. In fact, in a longitudinal study with trained singers performing various slow and fast singing tasks, vocal accuracy was not differentially affected by masked auditory feedback neither before nor after 3 years of vocal training (Mürbe et al., 2004), which suggests that auditory feedback may not play a crucial role in vocal accuracy after extensive vocal training. Nevertheless, vocal accuracy did improve during slow singing tasks with masked feedback after vocal training, which Mürbe et al. (2004) attributed to training-enhanced “neuromuscular memory of pitch” (p. 240). This implies that trained singers may rely more on somatosensory feedback to make sure that notes are produced properly, since they can still sing accurately for some time after losing their hearing (Wyke, 1974). Indeed, a functional magnetic resonance imaging (fMRI) singing study demonstrated that both vocal students (enrolled in a performance program) and professional opera singers recruited more activity within S1 and somatosensory association cortex than amateur singers, and moreover, the amount of singing practice positively correlated with the activity in these regions (Kleber et al., 2010). In a more recent fMRI study, Kleber et al. (2013) effectively reduced the amount of somatosensory feedback available by applying a topical anesthetic to the vocal folds just prior to singing in the MR scanner. The investigators determined that under vocal-fold anesthesia, singers displayed reduced activity in the right anterior insula than non-musicians, who had enhanced insular activity with anesthesia. Additionally, this region exhibited decreased functional connectivity to M1, S1, and auditory cortex in singers under topical anesthesia, while functional connectivity increased between these regions in non-musicians with anesthetized vocal folds. Notably, singers still sang more accurately under anesthesia than non-musicians, despite the observed reduction of insular activity and functional connectivity. Both of Kleber's experiments provide evidence that: (1) singers may rely more heavily on somatosensory feedback as a function of vocal training and practice, and (2) singers, perhaps by virtue of their training, can regulate activity within the right anterior insula to “disengage” or ignore somatosensory feedback when it is perturbed or deemed unreliable and thus may significantly alter their singing performance.

Similar to the somatosensory feedback perturbation induced in Kleber's recent study, Zarate and colleagues (2008, 2010b) utilized pitch-shifted auditory feedback with fMRI techniques to target explicitly the brain regions involved in auditory-vocal motor control in singing. As discussed earlier, pitch-altered feedback elicits pitch-shift responses that often contain early and late components. Larson and colleagues suggested that the early pitch-shift response, which may be governed by the midbrain PAG, is a more automatic reaction used to stabilize vocal output by correcting small, unexpected fluctuations in vocal pitch; the late pitch-shift response, on the other hand, may be under more voluntary control—perhaps controlled by the auditory cortex, ACC, etc.,—and thus may contribute to vocal pitch control during speaking and singing (Burnett et al., 1998; Larson, 1998; Hain et al., 2000; Liu and Larson, 2007). Indeed, although trained singers exhibit early pitch-shift responses to briefly pitch-shifted feedback, they were still able to maintain their intended goal for vocalization (either sustaining a steady pitch or glissandos, Burnett and Larson, 2002; Hafke, 2008), perhaps due to enhanced top–down control of the late pitch-shift response that resulted from years of vocal training. In contrast, non-musicians may not exhibit such precise vocal control over the late pitch-shift response. To assess the effects of extensive vocal training on pitch control in singing, Zarate and colleagues (2008, 2010b) tested singers and non-musicians with two singing tasks that required different types of top–down voluntary control: (1) an “ignore” task where subjects were required to hold their pitch steady, despite hearing pitch-shifted auditory feedback; and (2) a “compensate” task in which subjects had to voluntarily adjust their vocal pitch precisely to correct for the pitch shift. The authors hypothesized that ignoring a small pitch shift would not only elicit an early pitch-shift response, but also target the PAG relative to the compensate task, which was specifically designed to engage their proposed cortical substrates for auditory-motor control of vocal pitch—auditory cortex, insula, and ACC (Zarate and Zatorre, 2008; Zarate et al., 2010b).

**Figure 2:**
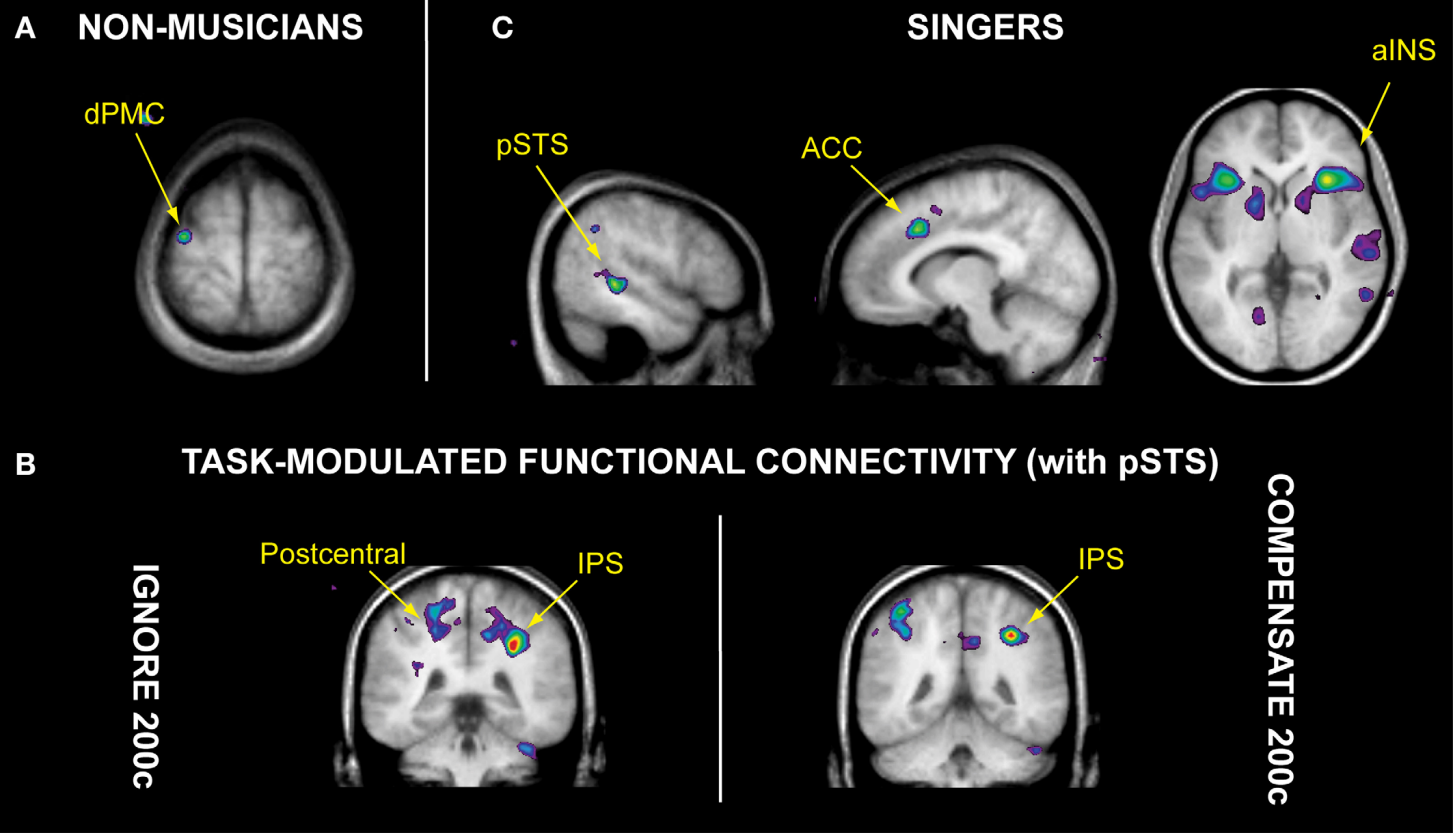
**Brain regions involved in auditory-motor control of singing, as observed in non-musicians and singers. (A)** When voluntarily correcting for a 200-cent pitch shift in auditory feedback (“compensate 200c” task), non-musicians recruited more activity within the dorsal premotor cortex (dPMC) than singers. **(B)** Singers engaged the posterior superior temporal sulcus (pSTS), anterior cingulate cortex (ACC), and anterior insula (aINS) when performing the “compensate 200c” task. **(C)** Analyses of task-modulated functional connectivity revealed that relative to singing with normal auditory feedback, the 200-cent pitch shift specifically enhanced functional connectivity between right pSTS and intraparietal sulcus (IPS) during both the “ignore 200c” and “compensate 200c” tasks, as well as the postcentral gyrus (containing somatosensory cortex) during the “ignore 200c” task. Data from Zarate and colleagues (2008, 2010b).

Due to the temporal limitations of fMRI methodology, Zarate et al. (2010b) were not able to determine whether the PAG is involved particularly with eliciting early pitch-shift responses, since these responses have a latency that is shorter than the best temporal resolution for fMRI. Nevertheless, two interesting cortical findings from their singing tasks were observed. First, both groups recruited the IPS and dorsal premotor cortex (dPMC) in each pitch-shifted singing task, compared to singing with normal feedback (Zarate and Zatorre, 2008). The authors suggested that since the IPS is associated with transformations of sensory input for motor preparation (Astafiev et al., 2003; Grefkes et al., 2004; Tanabe et al., 2005), it was recruited specifically during transformations of auditory input (see Foster and Zatorre, 2010; Zatorre et al., 2010; Foster et al., 2013) into spatial information within the frequency domain (i.e., up or down). This “frequency spatial information” can then be used by the dPMC—an area that receives indirect connections from auditory and parietal areas via the insula (Mufson and Mesulam, 1982), and is attributed to conditional sensory-motor associations (Petrides, 1986; Chouinard and Paus, 2006)—to prepare a vocal response (e.g., maintain steady vocal output or correct for the pitch shift). Second, despite the observed lack of performance differences in the compensate task—i.e., both groups voluntarily adjusted for the pitch-shifted feedback to a similar extent—different neural substrates for auditory-motor control were recruited in each group. Compared to singers, the non-musicians exhibited more activity within the dPMC while voluntarily correcting for the pitch shift (Figure 2A; Zarate and Zatorre, 2008); the authors proposed that the dPMC was recruited selectively in non-musicians as they learned to associate a pitch-shift “cue” in auditory feedback with a corrective adjustment in vocal pitch. Therefore, this region may constitute a basic substrate for voluntary auditory-motor control of vocal pitch (Zarate and Zatorre, 2008) and perhaps music production in general—after more training and practice, the dPMC is recruited less in non-musicians during the same musical production task that was learned (and assessed with fMRI) at earlier stages of an experiment (Chen et al., 2012). Indeed, rather than recruiting the dPMC, singers engaged auditory cortex within the pSTS, anterior insula, and ACC for this task (Figure 2B; Zarate and Zatorre, 2008; Zarate et al., 2010b). Moreover, voluntary vocal-control singing tasks (i.e., compensating for and ignoring large pitch shifts in feedback) specifically enhanced the functional connectivity between the pSTS and IPS (Figure 2C; Zarate et al., 2010b). Given the IPS' role in sensory-motor transformations, Zarate and colleagues suggested that within singers, the auditory cortex and IPS jointly process and extract pitch-shift information that can be used to control vocal pitch (e.g., magnitude and direction of the pitch shift). Since the auditory cortex is functionally connected to the insula and ACC (Zarate and Zatorre, 2008; Zarate et al., 2010b), the pitch-shift information may be sent via the anterior insula to the ACC for initiation of the task-appropriate vocal motor program (i.e., maintain the originally produced note or correct for the shift). The authors proposed that these four cortical regions constitute an experience-dependent network for auditory-motor control of the singing voice, which may be recruited increasingly as a function of more vocal training and practice.

### Short-term training effects on auditory and vocal skills and their neural correlates

Based on the studies above, trained singers may have more precise vocal control compared to non-musicians, due to extensive vocal training that recruits an experience-dependent cortical network and/or selectively gates access to sensory feedback within this network. However, Amir et al. (2003) determined that instrumental musicians (without formal vocal training) also sang more accurately than non-musicians in a simple pitch-matching task, in which subjects were required to sing a note that was just presented. Additionally, two studies report a significant correlation between pitch discrimination and vocal accuracy in both instrumental musicians and non-musicians—individuals who sang more accurately also had better discrimination skills (Amir et al., 2003; Watts et al., 2005). If this observed correlational relationship is a causal one, as these studies suggest, then refining pitch-discrimination skills may lead to better vocal accuracy. For instance, many studies have reported that auditory training improves pitch discrimination both at the training frequency and at other non-trained frequencies (Demany, 1985; Delhommeau et al., 2002, 2005; Ari-Even Roth et al., 2003). Furthermore, the effects of auditory training with pure tones also generalize to more complex tones (Grimault et al., 2003). In light of these observations and the proposed causal relationship between pitch discrimination and vocal accuracy, the newly enhanced ability to discriminate between pitches (following training) may increase the likelihood of detecting slight errors in vocal output, which may result in increased vocal accuracy. In turn, these training-induced behavioral changes are often accompanied by neural plasticity. For example, after non-musicians had received pitch-discrimination training, improved pitch discrimination was accompanied by enhanced auditory cortical responses (Bosnyak et al., 2004). Additionally, when non-musicians were trained to associate specific piano keys with their corresponding pitches and play short piano melodies, significant training-induced increases in cortical activity were observed within auditory, sensorimotor, frontal, and parietal regions (Bangert and Altenmüller, 2003; Lahav et al., 2007).

Therefore, to examine whether: (1) singing accuracy improves subsequent to auditory training, and (2) auditory-training enhanced singing specifically engaged the experience-dependent network for auditory-motor control in singing (i.e., auditory cortex, IPS, anterior insula, and ACC), Zarate et al. (2010a) tested two groups of non-musicians—an experimental group that received training to improve their auditory discrimination skills, and a control group that received no training—with auditory discrimination and singing tasks. In this study, the investigators employed more naturalistic melodic singing tasks to target the experience-dependent network, since accurate production of novel melodies requires auditory-motor control in a similar fashion as voluntarily correcting for pitch-shifted feedback; the auditory feedback of the currently produced note may be monitored in order to produce the correct pitch interval to the next note. Although the experimental group displayed enhanced auditory discrimination skills and training-induced changes in auditory task-associated neural activity (Zatorre et al., 2012), they did not show significant improvements in singing performance or recruit the experience-dependent network for auditory-motor control in singing (Zarate et al., 2010a). Consequently, Zarate et al. (2010a) concluded that auditory training alone (at least in an experimental setting) is not sufficient to improve vocal performance or recruit the experience-dependent network for auditory-motor control of singing (auditory cortex, IPS, anterior insula, and ACC); perhaps only simultaneous enhancements in both auditory and vocal motor skills via extensive training (e.g., voice lessons) would bring forth improvements in vocal performance and engage this particular network.

## Sensory-motor control of singing in other populations

### Acquired vocal amusia

Clinical evidence that complements the proposed roles of the auditory cortex, IPS, S1, insula, and premotor regions during singing comes from case reports of brain lesions that result in vocal amusia or oral-expressive amusia (for a review, see Berkowska and Dalla Bella, 2009; Stewart et al., 2009). For instance, a woman with cortical atrophy in the right temporal lobe and insula, as well as diminished blood flow to right frontal and temporal regions, exhibited signs of progressive amusia and aprosodia—she gradually was incapable of perceiving and producing well-known melodies and affective intonation or prosody in speech (Confavreux et al., 1992). Additionally, a female tango singer who suffered a right-lateralized cerebral infarction presented with damage to right Heschl's gyrus and STG, inferior parietal regions including supramarginal gyrus and S1, and posterior insula; her music perception was greatly diminished post-stroke (relative to speech discrimination), and her singing was considered less stable within single notes, less accurate in pitch, and monotonous in affect (Terao et al., 2006).

While the two previous cases with damage to auditory cortex, insula, and other regions within the singing network presented with deficits in both music perception and production, two additional cases present perhaps the strongest evidence for these regions' involvement specifically for singing in the absence of impaired auditory perception. In a female patient who suffered a stroke in the right hemisphere affecting the lateral frontal lobe and M1, STG, insula, S1, and inferior parietal lobe, investigators observed impaired affective intonation in speech and the inability to sing pitch intervals accurately, while familiar-song perception and singing rhythms or melodic contour were relatively preserved (Murayama et al., 2004). Finally, a male amateur singer with right-lateralized damage to his posterior temporal lobe, inferior parietal lobe, insula, and inferior frontal gyrus presented with relatively spared speech comprehension and production, prosodic perception and production, music perception, and rhythm production; however, he exhibited specifically impaired pitch-interval production (Schön et al., 2004). This rather pure case of vocal amusia—in the absence of aphasia, aprosodia, and “perceptual” amusia—demonstrates that the damaged brain regions, which overlap with the areas outlined by Zarate and colleagues (2008, 2010b), contribute to the finely-grained sensory-motor control of singing.

### Congenital amusia

Recall that the same neural network is recruited for singing in healthy individuals, irrespective of the amount of vocal training or experience (see section Neuroimaging Evidence: A General Functional Network For Human Vocalization). However, when pitch processing is compromised as observed in congenital amusia (Ayotte et al., 2002; Peretz and Hyde, 2003; Foxton et al., 2004)—due to cortical malformations in the STG and inferior frontal gyrus (Hyde et al., 2007) and disrupted structural and functional connectivity (Loui et al., 2009; Hyde et al., 2011)—it may be assumed that pitch production in singing would similarly be affected as well. Yet, as observed in Murayama's et al. (2004) and Schön's et al. (2004) case reports, a dissociation between pitch perception and production skills can exist—following a stroke, spared pitch perception does not necessarily preclude inaccurate pitch production. Conversely, some individuals with congenital amusia still can sing pitch changes in the correct direction (e.g., up vs. down), match target notes, and sing familiar song excerpts somewhat accurately, despite observed problems with pitch perception (Ayotte et al., 2002; Loui et al., 2008; Dalla Bella et al., 2009; Hutchins et al., 2010).

Based on this behavioral evidence, as well as observations of singing in the general population, Berkowska and Dalla Bella proffered a “vocal sensorimotor loop” model to outline two functional pathways within the song system that may explain observations of accurate-pitch and poor-pitch singing (Berkowska and Dalla Bella, 2009; Dalla Bella et al., 2011). In this model, the authors list potential brain regions—based on previous neuroimaging studies, many of which are included in the section Neuroimaging Evidence: A General Functional Network For Human Vocalization—that contribute to mechanims underlying singing, such as: regions within the STG for processing auditory input, which includes the auditory target to be reproduced and auditory feedback; dorsal prefrontal cortex, inferior sensorimotor cortex, area “Spt” within the planum temporale, and insula for auditory-motor mapping and memory access; supplementary motor area, ACC, and insula for motor preparation; and ventral M1 for vocal motor execution. Berkowska and colleagues also make distinctions between two pathways—a covert pathway involved in pitch discrimination (that can be compromised in congenital amusia), and an overt pathway involved in pitch production—but they do not clarify which of the aforementioned brain regions belong to each pathway. Congenital amusia may be due to a structural and functional “disconnection” between right auditory and inferior frontal cortical regions that contribute to pitch processing—although the right auditory cortex exhibits differential responses to pitch changes, the right inferior frontal cortex does not show a correlated increase in activity, as it does in normal listeners (Hyde et al., 2011). Even though this particular covert pathway is affected, auditory input (e.g., presented auditory targets, auditory feedback, etc.) can still be processed by auditory cortex (Moreau et al., 2009; Peretz et al., 2009; Moreau et al., 2013). Hypothetically speaking, auditory input may then be processed further by IPS (depending on the amount of vocal training), anterior insula, and premotor regions (dPMC or ACC) for auditory-motor control of singing based on Zarate's findings (Zarate and Zatorre, 2008; Zarate et al., 2010b), rendering vocal production relatively spared in some instances of congenital amusia.

## Comparisons with models of auditory processing

**Figure 3:**
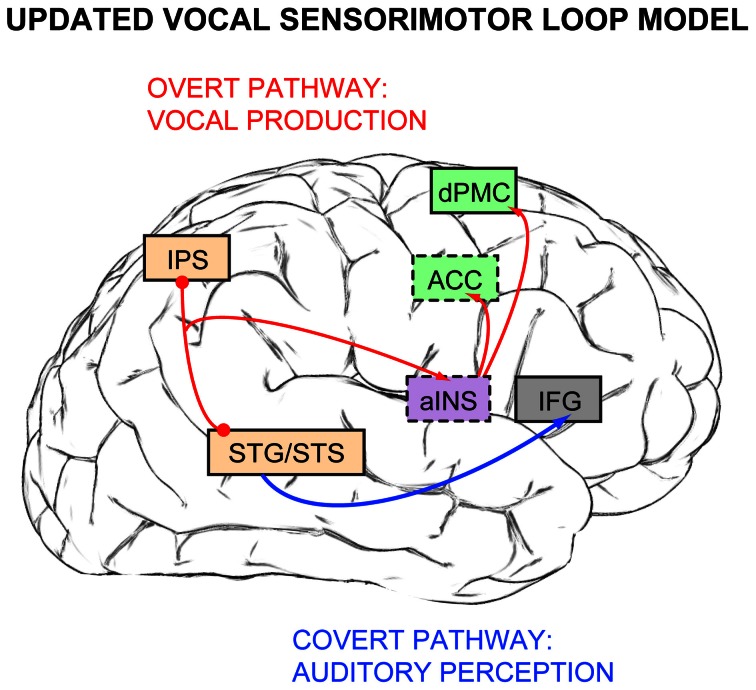
**A revised version of Berkowska and Dalla Bella's, Dalla Bella, and colleagues' (2009, 2011) vocal sensorimotor loop model for singing, updated with findings from Zarate and colleagues (2008, 2010b) fMRI studies.** The covert pathway for pitch production (blue arrow) includes auditory cortex and inferior frontal gyrus (IFG), while the overt pathway for vocal pitch production (red arrows) is comprised of auditory cortex (STG/STS), intraparietal sulcus (IPS), anterior insula (aINS), anterior cingulate cortex (ACC), and dorsal premotor cortex (dPMC). Brain regions that are not visible normally from this lateral brain view are indicated in boxes outlined with dashes. Box colors are retained from Figure 1: light orange for auditory processing, green for vocal motor control, purple for multimodal processing.

Berkowska and Dalla Bella's (2009), Dalla Bella et al.'s (2011) vocal sensorimotor loop model for singing, when enriched with neuroimaging evidence from Zarate and Zatorre (2008), Hyde et al. (2011), and Loui et al. (2009), potentially consists of auditory and inferior frontal cortex in the covert perception pathway (Figure 3, blue arrow), and auditory cortex, IPS, anterior insula, and premotor areas in the overt production pathway (Figure 3, red arrows). These updated pathways resemble the more recognized (and widely debated) dual-stream model for auditory processing, which was first proposed by Rauschecker and Tian (2000). The dorsal stream was originally suggested to be specialized for processing auditory spatial information (the “where” pathway), while the ventral stream was attributed with processing auditory object/sound identity information (the “what” pathway). The scientific debate focuses mostly on competing accounts and hypotheses of the dorsal stream's contributions, which include: (1) processing spectral changes over time (the “where in frequency” or “how” pathway, Belin and Zatorre, 2000); (2) extracting relevant sound features and matching them with stored templates of motor responses (the “do” pathway, Warren et al., 2005); (3) transforming auditory representations of speech into motor programs for speech gestures (Hickok and Poeppel, 2000, 2004, 2007); and (4) comparing between feedforward and feedback mechanisms (Rauschecker and Scott, 2009).

For our purposes here, the most relevant dorsal-stream models are the spectrotemporal processing account from Belin and Zatorre (2000) and auditory-motor transformation hypotheses for auditory spatial processing and speech from Warren et al. (2005) and Hickok and Poeppel (2000, 2004, 2007). It should be noted, however that the auditory-motor control network for singing conflicts with the latter two models, in which area Spt in the planum temporale is the sole neural substrate for auditory-motor transformations (Hickok and Poeppel, 2000, 2004; Warren et al., 2005; Hickok and Poeppel, 2007). Zarate's singing research (2008, 2010b) provides empirical evidence both supporting, and perhaps, updating these dorsal-stream models—auditory cortex and IPS process and extract pitch changes from feedback, and the pitch information is sent from these regions via the insula to premotor areas for vocal motor adjustments. Therefore, according to these neuroimaging findings, transformations of task-relevant auditory features into subsequent motor responses may not take place in only one brain region, as purported by the Warren et al. and Hickok/Poeppel models, but rather may be parceled among a network of different areas within the dorsal auditory stream. Thus, it could be argued that many brain regions along the dorsal auditory stream are involved in processing “how” auditory features change over time before executing or “doing” a specific motor act in response to these auditory events, regardless of the particular modality—be it information related to auditory space, speech, or music.

## Conclusion

**Figure 4:**
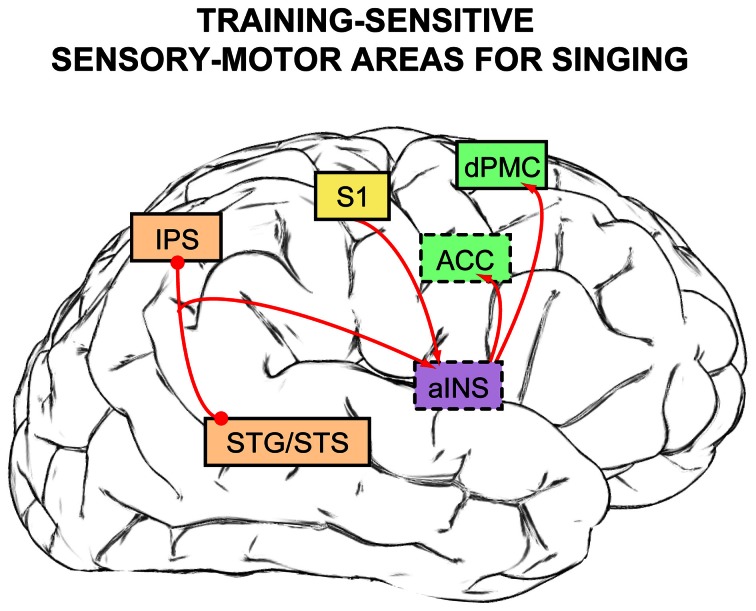
**Neural substrates for sensory-motor control of singing that are sensitive to the amount of vocal training [based on findings from Kleber et al. (2010, 2013), Zarate and Zatorre (2008), Zarate et al. (2010b)].** Brain regions that are not visible normally from this lateral brain view are indicated in boxes outlined with dashes, and box colors are retained from Figures 1 and 3. Activity within primary somatosensory cortex (S1) increases as a function of the amount of weekly vocal practice, suggesting a greater reliance on somatosensory feedback with more training and experience. After extensive vocal training and practice, the anterior insula (aINS) can serve a gating function for somatosensory feedback. Features within auditory feedback are processed and extracted by auditory cortex (STG/STS) and the intraparietal sulcus (IPS), and task-relevant auditory information is sent via the aINS to the dorsal premotor cortex (dPMC)—in people with little to no formal vocal training—or to the anterior cingulate cortex (ACC) in experienced singers to voluntarily adjust vocal output according to the singing task demands.

In this review, findings from over 20 years of research have been reviewed to outline a general neural network for song and speech production (section Neuroimaging Evidence: A General Functional Network For Human Vocalization). Within this functional network, cortical substrates that are specific for the sensory-motor control of singing pitch and are sensitive to the amount of vocal training have been identified (Figure 4): the pSTS and IPS for auditory processing and transformation for motor output (light orange boxes), S1 for somatosensory processing (yellow box), anterior insula (in purple, both for auditory-motor integration and somatosensory feedback gating), and premotor regions for vocal motor preparation and response initiation (dPMC and ACC, in green). When the auditory-related findings are placed within a larger framework—a dual-pathway (i.e., perception vs. production), sensory-motor model for singing (Berkowska and Dalla Bella, 2009)—these music-specific findings can then be linked to broader research interests in auditory cognition, such as auditory spatial localization and speech perception/production, due to the auditory-motor control network's similarity to prevalent dual-stream models of auditory processing as a whole.

### Conflict of interest statement

The author declares that the research was conducted in the absence of any commercial or financial relationships that could be construed as a potential conflict of interest.

## References

[B1] Ackermann H. Riecker A. (2004). The contribution of the insula to motor aspects of speech production: a review and a hypothesis. Brain Lang. 89, 320–328 10.1016/S0093-934X(03)00347-X 15068914

[B2] Ackermann H. Riecker A. (2010). The contribution(s) of the insula to speech production: a review of the clinical and functional imaging literature. Brain Struct. Funct. 214, 419–433 10.1007/s00429-010-0257-x 20512374

[B3] Ackermann H. Vogel M. Petersen D. Poremba M. (1992). Speech deficits in ischaemic cerebellar lesions. J. Neurol. 239, 223–227 1597689 10.1007/BF00839144

[B4] Alm P. A. (2004). Stuttering and the basal ganglia circuits: a critical review of possible relations. J. Commun. Disord. 37, 325–369 10.1016/j.jcomdis.2004.03.001 15159193

[B5] Amir O. Amir N. Kishon-Rubin L. (2003). The effect of superior auditory skills on vocal accuracy. J. Acoust. Soc. Am. 113, 1102–1108 10.1121/1.1536632 12597203

[B6] Anstis S. M. Cavanagh P. (1979). Adaptation to frequency-shifted auditory feedback. Percept. Psychophys. 26, 449–458 10.3758/BF03204284 542360

[B7] Ari-Even Roth D. Amir O. Alaluf L. Buchsenspanner S. Kishon-Rabin L. (2003). The effect of training on frequency discrimination: generalization to untrained frequencies and to the untrained ear. J. Basic Clin. Physiol. Pharmacol. 14, 137–150 14558728 10.1515/jbcpp.2003.14.2.137

[B8] Astafiev S. V. Shulman G. L. Stanley C. M. Snyder A. Z. Van E. D. C. Corbetta M. (2003). Functional organization of human intraparietal and frontal cortex for attending, looking, and pointing. J. Neurosci. 23, 4689–4699 12805308 10.1523/JNEUROSCI.23-11-04689.2003 PMC6740811

[B9] Augustine J. R. (1996). Circuitry and functional aspects of the insular lobe in primates including humans. Brain Res. Brain Res. Rev. 22, 229–244 10.1016/S0165-0173(96)00011-2 8957561

[B10] Ayotte J. Peretz I. Hyde K. (2002). Congenital amusia: a group study of adults afflicted with a music-specific disorder. Brain 125, 238–251 10.1093/brain/awf028 11844725

[B11] Bamiou D. E. Musiek F. E. Luxon L. M. (2003). The insula (Island of Reil) and its role in auditory processing. Literature review. Brain Res. Brain Res. Rev. 42, 143–154 10.1016/S0165-0173(03)00172-3 12738055

[B12] Bangert M. W. Altenmüller E. O. (2003). Mapping perception to action in piano practice: a longitudinal DC-EEG study. BMC Neurosci. 4:26 10.1186/1471-2202-4-26 14575529 PMC270043

[B13] Barbas H. Ghashghaei H. Dombrowski S. M. Rempel-Clower N. L. (1999). Medial prefrontal cortices are unified by common connections with superior temporal cortices and distinguished by input from memory-related areas in the rhesus monkey. J. Comp. Neurol. 410, 343–367 10.1002/(SICI)1096-9861(19990802)410:3<343::AID-CNE1>3.0.CO;2-1 10404405

[B14] Belin P. Zatorre R. J. (2000). ‘What’, ‘where’ and ‘how’ in auditory cortex. Nat. Neurosci. 3, 965–966 10.1038/79890 11017161

[B15] Belin P. Zatorre R. J. Lafaille P. Ahad P. Pike B. (2000). Voice-selective areas in human auditory cortex. Nature 403, 309–312 10.1038/35002078 10659849

[B16] Bendor D. Wang X. (2006). Cortical representations of pitch in monkeys and humans. Curr. Opin. Neurobiol. 16, 391–399 10.1016/j.conb.2006.07.001 16842992 PMC4325365

[B17] Berkowska M. Dalla Bella S. (2009). Acquired and congenital disorders of sung performance: a review. Adv. Cogn. Psychol. 5, 69–83 10.2478/v10053-008-0068-2 20523851 PMC2865000

[B18] Bosnyak D. J. Eaton R. A. Roberts L. E. (2004). Distributed auditory cortical representations are modified when non-musicians are trained at pitch discrimination with 40 Hz amplitude modulated tones. Cereb. Cortex 14, 1088–1099 10.1093/cercor/bhh068 15115745

[B19] Brown S. Martinez M. J. Hodges D. A. Fox P. T. Parsons L. M. (2004). The song system of the human brain. Brain Res. Cogn. Brain Res. 20, 363–375 10.1016/j.cogbrainres.2004.03.016 15268914

[B20] Brown S. Martinez M. J. Parsons L. M. (2006). Music and language side by side in the brain: a PET study of the generation of melodies and sentences. Eur. J. Neurosci. 23, 2791–2803 10.1111/j.1460-9568.2006.04785.x 16817882

[B21] Burnett T. A. Freedland M. B. Larson C. R. Hain T. C. (1998). Voice F0 responses to manipulations in pitch feedback. J. Acoust. Soc. Am. 103, 3153–3161 9637026 10.1121/1.423073

[B22] Burnett T. A. Larson C. (2002). Early pitch-shift response is active in both steady and dynamic voice pitch control. J. Acoust. Soc. Am. 112, 1058–1063 12243154 10.1121/1.1487844

[B23] Chartrand J. P. Belin P. (2006). Superior voice timbre processing in musicians. Neurosci. Lett. 405, 164–167 10.1016/j.neulet.2006.06.053 16860471

[B24] Chen J. L. Rae C. Watkins K. E. (2012). Learning to play a melody: an fMRI study examining the formation of auditory-motor associations. Neuroimage 59, 1200–1208 10.1016/j.neuroimage.2011.08.012 21871571

[B25] Chouinard P. A. Paus T. (2006). The primary motor and premotor areas of the human cerebral cortex. Neuroscientist 12, 143–152 10.1177/1073858405284255 16514011

[B26] Confavreux C. Croisile B. Garassus P. Aimard G. Trillet M. (1992). Progressive amusia and aprosody. Arch. Neurol. 49, 971–976 10.1001/archneur.1992.00530330095023 1520089

[B27] Dalla Bella S. Berkowska M. Sowiñski J. (2011). Disorders of pitch production in tone deafness. Front. Psychol. 2, 1–11 10.3389/fpsyg.2011.00164 21811479 PMC3140645

[B28] Dalla Bella S. Giguere J. F. Peretz I. (2009). Singing in congenital amusia. J. Acoust. Soc. Am. 126, 414–424 10.1121/1.3132504 19603898

[B29] Delhommeau K. Micheyl C. Jouvent R. (2005). Generalization of frequency discrimination learning across frequencies and ears: implications for underlying neural mechanisms in humans. J. Assoc. Res. Otolaryngol. 6, 171–179 10.1007/s10162-005-5055-4 15834630 PMC2538333

[B30] Delhommeau K. Micheyl C. Jouvent R. Collet L. (2002). Transfer of learning across durations and ears in auditory frequency discrimination. Percept. Psychophys. 64, 426–436 10.3758/BF03194715 12049283

[B31] Demany L. (1985). Perceptual learning in frequency discrimination. J. Acoust. Soc. Am. 78, 1118–1120 4031256 10.1121/1.393034

[B32] Dujardin E. Jürgens U. (2005). Afferents of vocalization-controlling periaqueductal regions in the squirrel monkey. Brain Res. 1034, 114–131 10.1016/j.brainres.2004.11.048 15713263

[B33] Esposito A. Demeurisse G. Alberti B. Fabbro F. (1999). Complete mutism after midbrain periaqueductal gray lesion. Neuroreport 10, 681–685 10208530 10.1097/00001756-199903170-00004

[B34] Formby C. Thomas R. G. Halsey J. H. Jr. (1989). Regional cerebral blood flow for singers and nonsingers while speaking, singing, and humming a rote passage. Brain Lang. 36, 690–698 2720376 10.1016/0093-934x(89)90094-1

[B35] Foster N. E. V. Zatorre R. J. (2010). A role for the intraparietal sulcus in transforming musical pitch information. Cereb. Cortex 20, 1350–1359 10.1093/cercor/bhp199 19789184

[B36] Foster N. E. V. Halpern A. R. Zatorre R. J. (2013). Common parietal activation in musical mental transformations across pitch and time. Neuroimage 75, 27–35 10.1016/j.neuroimage.2013.02.044 23470983

[B37] Foxton J. M. Dean J. L. Gee R. Peretz I. Griffiths T. D. (2004). Characterization of deficits in pitch perception underlying ‘tone deafness’. Brain 127, 801–810 10.1093/brain/awh105 14985262

[B38] Grabski K. Lamalle L. Vilain C. Schwartz J. L. Vallee N. Tropres I. (2012). Functional MRI assessment of orofacial articulators: neural correlates of lip, jaw, larynx, and tongue movements. Hum. Brain Mapp. 33, 2306–2321 10.1002/hbm.21363 21826760 PMC6870116

[B39] Grefkes C. Ritzl A. Zilles K. Fink G. R. (2004). Human medial intraparietal cortex subserves visuomotor coordinate transformation. Neuroimage 23, 1494–1506 10.1016/j.neuroimage.2004.08.031 15589113

[B40] Griffiths T. D. (2003). Functional imaging of pitch analysis. Ann. N.Y. Acad. Sci. 999, 40–49 10.1196/annals.1284.004 14681116

[B41] Griffiths T. D. Uppenkamp S. Johnsrude I. Josephs O. Patterson R. D. (2001). Encoding of the temporal regularity of sound in the human brainstem. Nat. Neurosci. 4, 633–637 10.1038/88459 11369945

[B42] Grimault N. Micheyl C. Carlyon R. P. Bacon S. P. Collet L. (2003). Learning in discrimination of frequency or modulation rate: generalization to fundamental frequency discrimination. Hear. Res. 184, 41–50 10.1016/S0378-5955(03)00214-4 14553902

[B44] Hafke H. Z. (2008). Nonconscious control of fundamental voice frequency. J. Acoust. Soc. Am. 123, 273–278 10.1121/1.2817357 18177157

[B45] Hain T. C. Burnett T. A. Kiran S. Larson C. R. Singh S. Kenney M. K. (2000). Instructing subjects to make a voluntary response reveals the presence of two components to the audio-vocal reflex. Exp. Brain Res. 130, 133–141 10.1007/s002219900237 10672466

[B46] Hannig S. Jürgens U. (2006). Projections of the ventrolateral pontine vocalization area in the squirrel monkey. Exp. Brain Res. 169, 92–105 10.1007/s00221-005-0128-5 16292643

[B47] Hardcastle W. J. (1976). Physiology of Speech Production: An Introduction for Speech Scientists. London: Academic Press, Ltd

[B48] Hickok G. Poeppel D. (2000). Towards a functional neuroanatomy of speech perception. Trends Cogn. Sci. 4, 131–138 10.1016/S1364-6613(00)01463-7 10740277

[B49] Hickok G. Poeppel D. (2004). Dorsal and ventral streams: a framework for understanding aspects of the functional anatomy of language. Cognition 92, 67–99 10.1016/j.cognition.2003.10.011 15037127

[B50] Hickok G. Poeppel D. (2007). The cortical organization of speech processing. Nat. Rev. Neurosci. 8, 393–402 10.1038/nrn2113 17431404

[B51] Hillis A. E. Work M. Barker P. B. Jacobs M. A. Breese E. L. Maurer K. (2004). Re-examining the brain regions crucial for orchestrating speech articulation. Brain 127, 1479–1487 10.1093/brain/awh172 15090478

[B52] Hirano M. Ohala J. Vennard W. (1969). The function of laryngeal muscles in regulating fundamental frequency and intensity of phonation. J. Speech Hear. Res. 12, 616–628 5811852 10.1044/jshr.1203.616

[B53] Houde J. F. Jordan M. I. (1998). Sensorimotor adaptation in speech production. Science 279, 1213–1216 10.1126/science.279.5354.1213 9469813

[B54] Houde J. F. Jordan M. I. (2002). Sensorimotor adaptation of speech I: compensation and adaptation. J. Speech Lang. Hear. Res. 45, 295–310 12003512 10.1044/1092-4388(2002/023)

[B55] Hudspeth A. J. (2000). Hearing, in Principles of Neural Science, eds Kandel E. R. Schwartz J. H. Jessel T. M. (New York, NY: McGraw-Hill), 590–613

[B56] Hutchins S. Zarate J. M. Zatorre R. J. Peretz I. (2010). An acoustical study of vocal pitch matching in congenital amusia. J. Acoust. Soc. Am. 127, 504–512 10.1121/1.3270391 20058995

[B57] Hyde K. L. Lerch J. P. Zatorre R. J. Griffiths T. D. Evans A. C. Peretz I. (2007). Cortical thickness in congenital amusia: when less is better than more. J. Neurosci. 27, 13028–13032 10.1523/JNEUROSCI.3039-07.2007 18032676 PMC6673307

[B58] Hyde K. L. Peretz I. Zatorre R. J. (2008). Evidence for the role of the right auditory cortex in fine pitch resolution. Neuropsychologia 46, 632–639 10.1016/j.neuropsychologia.2007.09.004 17959204

[B59] Hyde K. L. Zatorre R. J. Peretz I. (2011). Functional MRI evidence of an abnormal neural network for pitch processing in congenital amusia. Cereb. Cortex 21, 292–299 10.1093/cercor/bhq094 20494966

[B60] Jeffries K. J. Braun A. R. Fritz J. B. (2003). Words in melody: an H 2 15 O PET study of brain activation during singing and speaking. Neuroreport 14, 749–754 10.1097/01.wnr.0000066198.94941.a4 12692476

[B61] Johnsrude I. S. Penhune V. B. Zatorre R. J. (2000). Functional specificity in the right human auditory cortex for perceiving pitch direction. Brain 123, 155–163 10.1093/brain/123.1.155 10611129

[B62] Jones E. G. Powell T. P. S. (1970). Connexions of the somatic sensory cortex of the rhesus monkey: III.—thalamic connexions. Brain 93, 37–56 10.1093/brain/93.1.37 4984909

[B63] Jones J. A. Keough D. (2008). Auditory-motor mapping for pitch control in singers and nonsingers. Exp. Brain Res. 190, 279–287 10.1007/s00221-008-1473-y 18592224 PMC2644332

[B64] Jones J. A. Munhall K. G. (2000). Perceptual calibration of F0 production: evidence from feedback perturbation. J. Acoust. Soc. Am. 108, 1246–1251 11008824 10.1121/1.1288414

[B65] Jones J. A. Munhall K. G. (2005). Remapping auditory-motor representations in voice production. Curr. Biol. 15, 1768–1772 10.1016/j.cub.2005.08.063 16213825

[B66] Jürgens U. (1983). Afferent fibers to the cingular vocalization region in the squirrel monkey. Exp. Neurol. 80, 395–409 10.1016/0014-4886(83)90291-1 6840246

[B67] Jürgens U. (2002). Neural pathways underlying vocal control. Neurosci. Biobehav. Rev. 26, 235–258 10.1016/S0149-7634(01)00068-9 11856561

[B68] Jürgens U. (2009). The neural control of vocalization in mammals: a review. J. Voice 23, 1–10 10.1016/j.jvoice.2007.07.005 18207362

[B69] Jürgens U. Hage S. R. (2007). On the role of the reticular formation in vocal pattern generation. Behav. Brain Res. 182, 308–314 10.1016/j.bbr.2006.11.027 17173983

[B70] Jürgens U. Kirzinger A. (1985). The laryngeal sensory pathway and its role in phonation. A brain lesioning study in the squirrel monkey. Exp. Brain Res. 59, 118–124 4018191 10.1007/BF00237672

[B71] Jungblut M. Huber W. Pustelniak M. Schnitker R. (2012). The impact of rhythm complexity on brain activation during simple singing: an event-related fMRI study. Restor. Neurol. Neurosci. 30, 39–53 10.3233/RNN-2011-0619 22082766

[B72] Kishon-Rabin L. Amir O. Vexler Y. Zaltz Y. (2001). Pitch discrimination: are professional musicians better than non-musicians? J. Basic Clin. Physiol. Pharmacol. 12, 125–143 11605682 10.1515/jbcpp.2001.12.2.125

[B73] Kleber B. Birbaumer N. Veit R. Trevorrow T. Lotze M. (2007). Overt and imagined singing of an Italian aria. Neuroimage 36, 889–900 10.1016/j.neuroimage.2007.02.053 17478107

[B74] Kleber B. Veit R. Birbaumer N. Gruzelier J. Lotze M. (2010). The brain of opera singers: experience-dependent changes in functional activation. Cereb. Cortex 20, 1144–1152 10.1093/cercor/bhp177 19692631

[B75] Kleber B. Zeitouni A. Friberg A. Zatorre R. J. (2013). Experience-dependent modulation of feedback integration during singing: role of the right anterior insula. J. Neurosci. 33, 6070–6080 10.1523/JNEUROSCI.4418-12.2013 23554488 PMC6618920

[B76] Kriegstein K. V. Giraud A. L. (2004). Distinct functional substrates along the right superior temporal sulcus for the processing of voices. Neuroimage 22, 948–955 10.1016/j.neuroimage.2004.02.020 15193626

[B77] Lahav A. Saltzman E. Schlaug G. (2007). Action representation of sound: audiomotor recognition network while listening to newly acquired actions. J. Neurosci. 27, 308–314 10.1523/JNEUROSCI.4822-06.2007 17215391 PMC6672064

[B78] Larson C. R. (1998). Cross-modality influences in speech motor control: the use of pitch shifting for the study of F0 control. J. Commun. Disord. 31, 489–502 10.1016/S0021-9924(98)00021-5 9836138

[B79] Larson C. R. Burnett T. A. Kiran S. (2000). Effects of pitch-shift velocity on voice F0 response. J. Acoust. Soc. Am. 107, 559–564 10641664 10.1121/1.428323

[B80] Lewis J. W. Beauchamp M. S. Deyoe E. A. (2000). A comparison of visual and auditory motion processing in human cerebral cortex. Cereb. Cortex 10, 873–888 10.1093/cercor/10.9.873 10982748

[B81] Liu H. Larson C. R. (2007). Effects of perturbation magnitude and voice F0 level on the pitch-shift reflex. J. Acoust. Soc. Am. 122, 3671–3677 10.1121/1.2800254 18247774

[B82] Lombard E. (1911). Le signe de l'elevation de la voix. Annales maladies oreille larynx nez pharynx 37, 101–119

[B83] Loui P. Alsop D. Schlaug G. (2009). Tone deafness: a new disconnection syndrome? J. Neurosci. 29, 10215–10220 10.1523/JNEUROSCI.1701-09.2009 19692596 PMC2747525

[B84] Loui P. Guenther F. H. Mathys C. Schlaug G. (2008). Action-perception mismatch in tone-deafness. Curr. Biol. 18, R331–R332 10.1016/j.cub.2008.02.045 18430629 PMC2791531

[B85] Mesulam M. M. Mufson E. J. (1982). Insula of the old world monkey. III: efferent cortical output and comments on function. J. Comp. Neurol. 212, 38–52 10.1002/cne.902120104 7174907

[B86] Micheyl C. Delhommeau K. Perrot X. Oxenham A. J. (2006). Influence of musical and psychoacoustical training on pitch discrimination. Hear. Res. 219, 36–47 10.1016/j.heares.2006.05.004 16839723

[B87] Moreau P. Jolicoeur P. Peretz I. (2009). Automatic brain responses to pitch changes in congenital amusia. Ann. N.Y. Acad. Sci. 1169, 191–194 10.1111/j.1749-6632.2009.04775.x 19673779

[B88] Moreau P. Jolicoeur P. Peretz I. (2013). Pitch discrimination without awareness in congenital amusia: evidence from event-related potentials. Brain Cogn. 81, 337–344 10.1016/j.bandc.2013.01.004 23434917

[B89] Müller-Preuss P. Jürgens U. (1976). Projections from the ‘cingular’ vocalization area in the squirrel monkey. Brain Res. 103, 29–43 10.1016/0006-8993(76)90684-3 56207

[B90] Müller-Preuss P. Newman J. D. Jürgens U. (1980). Anatomical and physiological evidence for a relationship between the ‘cingular’ vocalization area and the auditory cortex in the squirrel monkey. Brain Res. 202, 307–315 10.1016/0006-8993(80)90143-2 7437905

[B91] Mürbe D. Pabst F. Hofmann G. Sundberg J. (2004). Effects of a professional solo singer education on auditory and kinesthetic feedback–a longitudinal study of singers' pitch control. J. Voice 18, 236–241 10.1016/j.jvoice.2003.05.001 15193657

[B92] Mufson E. J. Mesulam M. M. (1982). Insula of the old world monkey. II: afferent cortical input and comments on the claustrum. J.Comp. Neurol. 212, 23–37 10.1002/cne.902120103 7174906

[B93] Murayama J. Kashiwagi T. Kashiwagi A. Mimura M. (2004). Impaired pitch production and preserved rhythm production in a right brain-damaged patient with amusia. Brain Cogn. 56, 36–42 10.1016/j.bandc.2004.05.004 15380874

[B94] Nonaka S. Takahashi R. Enomoto K. Katada A. Unno T. (1997). Lombard reflex during PAG-induced vocalization in decerebrate cats. Neurosci. Res. 29, 283–289 10.1016/S0168-0102(97)00097-7 9527619

[B95] Owren M. J. Amoss R. T. Rendall D. (2011). Two organizing principles of vocal production: implications for nonhuman and human primates. Am. J. Primatol. 73, 530–544 10.1002/ajp.20913 21509789

[B96] Özdemir E. Norton A. Schlaug G. (2006). Shared and distinct neural correlates of singing and speaking. Neuroimage 33, 628–635 10.1016/j.neuroimage.2006.07.013 16956772

[B97] Patterson R. D. Uppenkamp S. Johnsrude I. S. Griffiths T. D. (2002). The processing of temporal pitch and melody information in auditory cortex. Neuron 36, 767–776 10.1016/S0896-6273(02)01060-7 12441063

[B98] Paus T. Petrides M. Evans A. C. Meyer E. (1993). Role of the human anterior cingulate cortex in the control of oculomotor, manual, and speech responses: a positron emission tomography study. J. Neurophysiol. 70, 453–469 8410148 10.1152/jn.1993.70.2.453

[B99] Penfield W. Rasmussen T. (1950). The Cerebral Cortex of Man: A Clinical Study of Localization of Function. New York, NY: MacMillan Co

[B101] Peretz I. Brattico E. Järvenpää M. Tervaniemi M. (2009). The amusic brain: in tune, out of key, and unaware. Brain 132, 1277–1286 10.1093/brain/awp055 19336462

[B102] Peretz I. Hyde K. L. (2003). What is specific to music processing? Insights from congenital amusia. Trends Cogn. Sci. 7, 362–367 10.1016/S1364-6613(03)00150-5 12907232

[B103] Perkell J. S. (2012). Movement goals and feedback and feedforward control mechanisms in speech production. J. Neurolinguistics 25, 382–407 10.1016/j.jneuroling.2010.02.011 22661828 PMC3361736

[B104] Perry D. W. Zatorre R. J. Petrides M. Alivisatos B. Meyer E. Evans A. C. (1999). Localization of cerebral activity during simple singing. Neuroreport 10, 3979–3984 10716244 10.1097/00001756-199912160-00046

[B105] Petrides M. (1986). The effect of periarcuate lesions in the monkey on the performance of symmetrically and asymmetrically reinforced visual and auditory go, no-go tasks. J. Neurosci. 6, 2054–2063 3734876 10.1523/JNEUROSCI.06-07-02054.1986 PMC6568599

[B106] Poeppel D. Guillemin A. Thompson J. Fritz J. Bavelier D. Braun A. R. (2004). Auditory lexical decision, categorical perception, and FM direction discrimination differentially engage left and right auditory cortex. Neuropsychologia 42, 183–200 10.1016/j.neuropsychologia.2003.07.010 14644105

[B107] Purcell D. W. Munhall K. G. (2006a). Adaptive control of vowel formant frequency: evidence from real-time formant manipulation. J. Acoust. Soc. Am. 120, 966–977 16938984 10.1121/1.2217714

[B108] Purcell D. W. Munhall K. G. (2006b). Compensation following real-time manipulation of formants in isolated vowels. J. Acoust. Soc. Am. 119, 2288–2297 16642842 10.1121/1.2173514

[B109] Rauschecker J. P. Scott S. K. (2009). Maps and streams in the auditory cortex: nonhuman primates illuminate human speech processing. Nat. Neurosci. 12, 718–724 10.1038/nn.2331 19471271 PMC2846110

[B110] Rauschecker J. P. Tian B. (2000). Mechanisms and streams for processing of “what” and “where” in auditory cortex. Proc. Natl. Acad. Sci. U.S.A. 97, 11800–11806 10.1073/pnas.97.22.11800 11050212 PMC34352

[B111] Riecker A. Ackermann H. Wildgruber D. Dogil G. Grodd W. (2000). Opposite hemispheric lateralization effects during speaking and singing at motor cortex, insula and cerebellum. Neuroreport 11, 1997–2000 10884059 10.1097/00001756-200006260-00038

[B112] Riecker A. Mathiak K. Wildgruber D. Erb M. Hertrich I. Grodd W. (2005). fMRI reveals two distinct cerebral networks subserving speech motor control. Neurology 64, 700–706 10.1212/01.WNL.0000152156.90779.89 15728295

[B113] Rivier F. Clarke S. (1997). Cytochrome oxidase, acetylcholinesterase, and NADPH-diaphorase staining in human supratemporal and insular cortex: evidence for multiple auditory areas. Neuroimage 6, 288–304 10.1006/nimg.1997.0304 9417972

[B114] Saito Y. Ishii K. Yagi K. Tatsumi I. F. Mizusawa H. (2006). Cerebral networks for spontaneous and synchronized singing and speaking. Neuroreport 17, 1893–1897 10.1097/WNR.0b013e328011519c 17179865

[B115] Schön D. Lorber B. Spacal M. Semenza C. (2004). A selective deficit in the production of exact musical intervals following right-hemisphere damage. Cogn. Neuropsychol. 21, 773–784 10.1080/02643290342000401 21038233

[B116] Schultz-Coulton H. J. (1978). The neuromuscular phonatory control system and vocal function. Acta Otolaryngol. 86, 142–153 696292 10.3109/00016487809124731

[B117] Schulz G. M. Varga M. Jeffires K. Ludlow C. L. Braun A. R. (2005). Functional neuroanatomy of human vocalization: an H215O PET study. Cereb. Cortex 15, 1835–1847 10.1093/cercor/bhi061 15746003

[B118] Simonyan K. Horwitz B. (2011). Laryngeal motor cortex and control of speech in humans. Neuroscientist 17, 197–208 10.1177/1073858410386727 21362688 PMC3077440

[B119] Stewart L. Von Kriegstein K. Dalla Bella S. Warren J. D. Griffiths T. D. (2009). Disorders of musical cognition, in Oxford Handbook of Music Psychology, eds Hallam S. Cross I. Thaut M. (New York, NY: Oxford University Press, Inc.), 184–196

[B120] Suga N. Yajima Y. (1988). Auditory-vocal integration in the midbrain of the mustached bat: periaqueductal gray and reticular formation, in The Physiological Control of Mammalian Vocalization, ed Newman J. D. (New York, NY: Plenum Press), 87–107

[B121] Sundberg J. (1987). The Science of the Singing Voice. DeKalb, IL: Northern Illinois University Press

[B122] Tanabe H. C. Kato M. Miyauchi S. Hayashi S. Yanagida T. (2005). The sensorimotor transformation of cross-modal spatial information in the anterior intraparietal sulcus as revealed by functional MRI. Brain Res. Cogn. Brain Res. 22, 385–396 10.1016/j.cogbrainres.2004.09.010 15722209

[B123] Terao Y. Mizuno T. Shindoh M. Sakurai Y. Ugawa Y. Kobayashi S. (2006). Vocal amusia in a professional tango singer due to a right superior temporal cortex infarction. Neuropsychologia 44, 479–488 10.1016/j.neuropsychologia.2005.05.013 15982678

[B124] Tervaniemi M. Just V. Koelsch S. Widmann A. Schroger E. (2005). Pitch discrimination accuracy in musicians vs nonmusicians: an event-related potential and behavioral study. Exp. Brain Res. 161, 1–10 10.1007/s00221-004-2044-5 15551089

[B125] Thoms G. Jürgens U. (1987). Common input of the cranial motor nuclei involved in phonation in squirrel monkey. Exp. Neurol. 95, 85–99 10.1016/0014-4886(87)90009-4 3792484

[B126] Warren J. D. Scott S. K. Price C. J. Griffiths T. D. (2006). Human brain mechanisms for the early analysis of voices. Neuroimage 31, 1389–1397 10.1016/j.neuroimage.2006.01.034 16540351

[B127] Warren J. E. Wise R. J. Warren J. D. (2005). Sounds do-able: auditory-motor transformations and the posterior temporal plane. Trends Neurosci. 28, 636–643 10.1016/j.tins.2005.09.010 16216346

[B128] Watts C. Moore R. McCaghren K. (2005). The relationship between vocal pitch-matching skills and pitch discrimination skills in untrained accurate and inaccurate singers. J. Voice 19, 534–543 10.1016/j.jvoice.2004.09.001 16301100

[B129] Watts C. Murphy J. Barnes-Burroughs K. (2003). Pitch matching accuracy of trained singers, untrained subjects with talented singing voices, and untrained subjects with nontalented singing voices in conditions of varying feedback. J. Voice 17, 185–194 12825651 10.1016/s0892-1997(03)00023-7

[B130] Willis W. D. (1986). Ascending somatosensory systems, in Spinal Afferent Processing, ed Yaksh T. L. (New York, NY: Plenum Press), 398–416

[B131] Wyke B. D. (1974). Laryngeal neuromuscular control systems in singing. A review of current concepts. Folia Phoniatr. (Basel) 26, 295–306 4609861 10.1159/000263791

[B132] Yoshida Y. Tanaka Y. Hirano M. Nakashima T. (2000). Sensory innervation of the pharynx and larynx. Am. J. Med. 108 Suppl. 4a, 51S–61S 10718453 10.1016/s0002-9343(99)00342-3

[B133] Zarate J. M. Delhommeau K. Wood S. Zatorre R. J. (2010a). Vocal accuracy and neural plasticity following micromelody-discrimination training. PLoS ONE 5:e11181 10.1371/journal.pone.0011181 20567521 PMC2887372

[B134] Zarate J. M. Wood S. Zatorre R. J. (2010b). Neural networks involved in voluntary and involuntary vocal pitch regulation in experienced singers. Neuropsychologia 48, 607–618 10.1016/j.neuropsychologia.2009.10.025 19896958

[B135] Zarate J. M. Zatorre R. J. (2008). Experience-dependent neural substrates involved in vocal pitch regulation during singing. Neuroimage 40, 1871–1887 10.1016/j.neuroimage.2008.01.026 18343163

[B136] Zatorre R. J. Delhommeau K. Zarate J. M. (2012). Modulation of auditory cortex response to pitch variation following training with microtonal melodies. Front. Psychol. 3, 1–17 10.3389/fpsyg.2012.00544 23227019 PMC3514543

[B137] Zatorre R. J. Evans A. C. Meyer E. (1994). Neural mechanisms underlying melodic perception and memory for pitch. J. Neurosci. 14, 1908–1919 8158246 10.1523/JNEUROSCI.14-04-01908.1994 PMC6577137

[B138] Zatorre R. J. Halpern A. R. Bouffard M. (2010). Mental reversal of imagined melodies: a role for the posterior parietal cortex. J. Cogn. Neurosci. 22, 775–789 10.1162/jocn.2009.21239 19366283

